# Percutaneous interventions in tricuspid valve disease: a new era in cardiac treatment

**DOI:** 10.1097/MS9.0000000000003437

**Published:** 2025-05-30

**Authors:** Tochukwu R. Nzeako, Chukwuka Elendu, Ali Moradi, Olawale Olanisa, Adekunle Kiladejo

**Affiliations:** a Christiana Care Hospital, Newark, Delaware, USA; b Federal University Teaching Hospital, Owerri, Nigeria; c Centre for Translational Medicine, Semmelweis University, Budapest, Hungary; d Trinity Health Grand Rapids, Grand Rapids, Michigan, USA; e Holy Name Medical Center, Teaneck, New Jersey, USA

**Keywords:** percutaneous interventions, right-sided heart failure, transcatheter tricuspid valve repair, tricuspid regurgitation, tricuspid valve disease

## Abstract

Tricuspid valve disease (TVD), primarily tricuspid regurgitation (TR), is increasingly recognized for its significant morbidity and mortality. Traditional management with medical therapy or high-risk surgical interventions leaves many patients untreated. The emergence of percutaneous interventions introduces a transformative, minimally invasive alternative for high-risk surgical candidates. Clinical trials such as TRILUMINATE have demonstrated the efficacy of devices like TriClip, achieving TR reduction to moderate or less in 86% of patients at 1 year, alongside improvements in functional capacity (6MWT) and quality of life (KCCQ scores). Real-world data from the TriValve registry revealed a 91% procedural success rate, marked improvements in the NYHA functional class, and a low complication profile. Long-term follow-up confirms sustained symptom relief, reduced edema, and improved exercise tolerance, with KCCQ scores increasing by 18–22 points. Survival benefits have also been reported, with a 35% 1-year mortality reduction compared to medical management. Although long-term data are limited, emerging transcatheter tricuspid valve replacement (TTVR) devices like EVOQUE show promising outcomes. Challenges persist in patient selection, particularly in congenital or complex structural abnormalities. Integrating advanced imaging techniques, such as 3D echocardiography, enhances procedural planning and outcome assessment. Ongoing trials like TRILUMINATE Pivotal aim to address evidence gaps, refine techniques, and expand indications. As percutaneous approaches evolve, they can revolutionize TVD management, improving survival, quality of life, and functional capacity in this high-risk population. Further research will solidify their role in contemporary cardiac care.

## Introduction and background

Tricuspid valve disease (TVD) is an increasingly recognized condition that significantly contributes to morbidity and mortality worldwide. The tricuspid valve, one of the heart’s four valves, regulates blood flow between the right atrium and right ventricle. Dysfunction of the tricuspid valve can result in tricuspid regurgitation (TR), where the valve fails to close properly, or tricuspid stenosis (TS), where narrowing obstructs blood flow. Among these, TR is the most prevalent, particularly secondary TR, which arises due to right ventricular dilation and annular enlargement caused by left-sided heart disease, pulmonary hypertension, or arrhythmias such as atrial fibrillation ^[1,2]^.

**HIGHLIGHTS**

- Percutaneous approaches offer safer alternatives to surgery.
- Early outcomes indicate improved quality of life and function.
- Broader access depends on training, awareness, and evidence.

Despite being historically referred to as the “forgotten valve,” the clinical importance of TVD has become evident in recent years. Moderate-to-severe TR is estimated to affect approximately 1.6 million people in the United States alone, with the prevalence rising with age^[3]^. Untreated TR leads to progressive right heart failure, systemic congestion, and ultimately, poor quality of life and survival.

Symptoms such as fatigue, edema, ascites, and jugular venous distension often manifest late in the disease course, contributing to diagnostic delays and missed opportunities for timely intervention^[4]^. Furthermore, the presence of TVD complicates outcomes in patients undergoing interventions for left-sided valvular diseases, underscoring the need for an integrated approach to management^[5]^.

Traditional management strategies for TVD have primarily revolved around medical therapy and surgical intervention. Medical management focuses on addressing symptoms of systemic congestion through diuretics and optimizing treatment for underlying conditions such as left-sided heart disease. However, medical therapy does not halt disease progression, leaving a significant unmet need^[6]^. Surgical repair or replacement of the tricuspid valve is typically reserved for severe, symptomatic cases or when TR coexists with other valvular pathologies requiring surgical intervention. Unfortunately, isolated tricuspid valve surgery is associated with high operative mortality, ranging from 8% to 10%, due to factors such as late presentation, advanced right ventricular dysfunction, and the presence of multiple comorbidities^[7]^. Consequently, many patients are deemed ineligible for surgery, creating a large cohort of individuals with untreated or undertreated diseases^[8]^.

The emergence of percutaneous interventions has introduced a transformative approach to the management of TVD, offering a minimally invasive alternative for patients who are high-risk surgical candidates. This paradigm shift is supported by advancements in device technology and imaging techniques that enable precise and effective interventions. Percutaneous strategies include tricuspid valve repair using edge-to-edge techniques, annuloplasty devices, and replacement with transcatheter tricuspid valve prostheses. Early clinical trials and registries have demonstrated promising results, with improvements in symptoms, functional capacity, and quality of life^[9,10]^.

Importantly, these procedures have shown acceptable safety profiles, with lower procedural risks than surgery, thereby expanding treatment options for a broader patient population^[11]^. The clinical adoption of percutaneous interventions reflects a broader shift in cardiac care towards minimally invasive therapies, prioritizing patient outcomes and quality of life. By addressing the unmet needs in TVD management, these innovations can reduce the burden of heart failure hospitalizations, improve long-term survival, and enhance the overall delivery of care. Continued research is essential to refine techniques, develop novel devices, and generate robust long-term data to guide clinical practice^[12-14]^.

## Data collection

We performed a systematic literature search using PubMed, Embase, Scopus, and Google Scholar databases to identify relevant studies published up to December 2024. Our review followed a narrative approach, incorporating clinical trials, observational studies, meta-analyses, systematic reviews, and expert consensus statements.

Keywords used in the search included “tricuspid valve disease,” “tricuspid regurgitation,” “percutaneous tricuspid valve repair,” “transcatheter tricuspid valve replacement,” “tricuspid annuloplasty,” and “minimally invasive cardiac interventions.” Inclusion criteria for this review encompassed peer-reviewed articles written in English that focused on the pathophysiology, clinical presentation, management strategies, and outcomes of percutaneous interventions in TVD. Studies addressing primary and secondary TVD and articles discussing emerging technologies and future directions in this field were included.

Exclusion criteria included non-English articles, studies with incomplete data, and those that solely addressed surgical approaches without mention of percutaneous interventions. Articles focusing on pediatric populations or congenital tricuspid valve abnormalities were excluded unless relevant to the broader discussion.

A total of 210 articles were initially identified through the keyword search. After screening titles and abstracts for relevance and applying the inclusion and exclusion criteria, 68 full-text articles were selected for detailed review. Of these, 52 met the final criteria and were included in this comprehensive analysis.

Data were extracted on study design, patient populations, types of percutaneous interventions, clinical outcomes, complications, and limitations. The findings were synthesized to provide a thorough understanding of the current landscape of percutaneous interventions in TVD, highlighting the paradigm shift they represent in cardiac care.

## Definition and epidemiology

TVD, often underdiagnosed and underappreciated, represents a significant global health burden, particularly due to its association with other cardiovascular pathologies. The prevalence of TVD varies widely, influenced by age, comorbidities, and diagnostic methods. Among the most common manifestations, TR affects approximately 1.6 million people in the United States alone, with moderate-to-severe cases observed in nearly 1.6% of the general population and up to 5% of individuals aged over 75 years^[1,2]^. These figures underscore the growing recognition of TVD as a critical component of cardiac care, particularly in aging populations.

Globally, TR is more prevalent than TS, with secondary or functional TR accounting for most cases. This subtype arises from right ventricular dilation and annular enlargement secondary to left-sided heart failure, pulmonary hypertension, or atrial fibrillation^[3]^. In contrast, primary TR, caused by structural abnormalities of the tricuspid valve apparatus, such as congenital malformations, infective endocarditis, or trauma, is relatively rare, comprising only 8% to 10% of all TR cases^[4]^.

The epidemiology of TVD reveals a significant gender disparity, with women disproportionately affected by TR. This trend may be linked to higher rates of rheumatic heart disease and mitral valve interventions in women, both of which can contribute to tricuspid annular dilation and subsequent regurgitation^[5]^. Furthermore, emerging data suggest that ethnic and geographic variations play a role in the prevalence of TVD. For instance, rheumatic tricuspid valve involvement remains a considerable concern in low- and middle-income countries, where rheumatic heart disease is endemic^[6]^. In high-income countries, however, secondary TR predominates, largely due to the increasing burden of heart failure and atrial fibrillation^[7]^.

Advancements in diagnostic imaging, particularly echocardiography, have enhanced the detection of TVD. Studies utilizing transthoracic and transesophageal echocardiography report a higher prevalence of asymptomatic or subclinical TR, emphasizing the importance of routine cardiovascular screening in at-risk populations^[8]^. Additionally, the advent of three-dimensional echocardiography and cardiac magnetic resonance imaging (MRI) has provided deeper insights into the pathophysiology and natural history of TVD, further refining its epidemiological understanding^[9]^.

The natural history of untreated TR is associated with significant morbidity and mortality. Severe TR, in particular, has been linked to progressive right-sided heart failure, reduced functional capacity, and poor long-term outcomes, even when left-sided cardiac conditions are addressed^[10]^. In a landmark cohort study, patients with severe TR demonstrated a 5-year survival rate of less than 40%, highlighting the urgent need for timely diagnosis and intervention^[11]^.

Emerging trends in the epidemiology of TVD include its increasing recognition in the context of transcatheter aortic valve replacement (TAVR) procedures. Post-TAVR TR has been identified as a common complication, affecting up to 25% of patients, with significant implications for prognosis and quality of life^[12]^. Similarly, TVD is gaining prominence in the era of advanced heart failure therapies, such as ventricular assist devices and cardiac transplantation, where tricuspid valve dysfunction often complicates clinical management^[13]^.

## Pathophysiology and clinical presentation of tricuspid valve disease

Anatomically, the tricuspid valve is named for its three primary leaflets: the anterior, posterior, and septal leaflets. The anterior leaflet is the largest and most prominent, closest to the right ventricular free wall. The posterior leaflet, while smaller, is situated near the right ventricular inflow region, and the septal leaflet is adjacent to the interventricular septum, connecting to the central fibrous body of the heart^[5,6]^. These leaflets form a dynamic structure that changes shape and configuration throughout the cardiac cycle to accommodate varying hemodynamic demands^[13,14]^.

The leaflets are tethered to the right ventricle by thin, fibrous cords called chordae tendineae, which prevent prolapse of the leaflets into the right atrium during systole. These chordae are connected to papillary muscles, which are muscular projections from the right ventricular myocardium. Typically, the right ventricle has three major papillary muscles: the anterior, posterior, and septal papillary muscles. These muscles contract during systole, tightening the chordae tendineae and ensuring proper coaptation of the valve leaflets^[13,14]^.

The tricuspid annulus forms the fibrous ring to which the leaflets are attached. This annular structure is not rigid; it is a dynamic and flexible ring that contracts and expands in synchrony with the cardiac cycle. The annulus plays a crucial role in maintaining the structural integrity and functionality of the tricuspid valve. The tricuspid annulus is elliptical in healthy individuals, with its major axis oriented along the anterior-posterior dimension. However, pathological conditions, such as right ventricular dilation or elevated pulmonary pressures, can lead to annular dilation and subsequent tricuspid valve dysfunction^[15,16]^.

Functionally, the tricuspid valve operates with the rest of the cardiac valves to regulate blood flow within the heart and ensure efficient circulation. During diastole, the valve opens passively, allowing deoxygenated blood from the right atrium to flow into the right ventricle. This process is facilitated by the pressure gradient between the atrium and ventricle, which promotes the unidirectional movement of blood. The tricuspid valve’s ability to open fully and maintain low resistance to flow is critical for optimizing right ventricular filling and cardiac output^[17]^.

During systole, when the right ventricle contracts to propel blood into the pulmonary artery, the tricuspid valve closes to prevent backflow into the right atrium. The coaptation of the valve leaflets, supported by the chordae tendineae and papillary muscles, ensures a tight seal and minimizes regurgitation. This synchronized mechanism is integral to maintaining forward flow and preventing the right atrium volume overload, which could otherwise lead to atrial dilation and subsequent complications^[18,19]^. The surrounding cardiac structures also influence the tricuspid valve’s function. The right atrium, ventricle, and interventricular septum provide the anatomical and functional framework supporting valve motion. The heart’s conduction system, particularly the atrioventricular node and bundle of His, is intimately related to the tricuspid valve’s annular region. This anatomical proximity has clinical significance, as interventions involving the tricuspid valve, such as surgical or percutaneous procedures, risk disrupting the conduction system and causing arrhythmias^[10,11]^.

Beyond its basic function, the tricuspid valve plays a role in adapting to physiological changes. For example, during exercise or increased cardiac output, the valve accommodates higher blood flow by allowing greater diastolic filling of the right ventricle. Conversely, the valve’s inability to maintain efficient function can lead to significant hemodynamic disturbances in pathological conditions, such as TR or stenosis.

TR, characterized by the backflow of blood from the right ventricle to the right atrium during systole, is the most common form of TVD and is classified as either primary or secondary (see Table 1). Primary TR results from structural abnormalities of the tricuspid valve apparatus, including leaflet defects, chordal rupture, or papillary muscle dysfunction^[20]^. These abnormalities may be congenital or acquired. Common congenital causes include Ebstein’s anomaly, characterized by apical displacement of the septal leaflet and atrialization of the right ventricle, leading to severe TR^[13]^. Acquired primary TR is often due to trauma, infective endocarditis, or rheumatic heart disease, which leads to leaflet thickening, scarring, and dysfunction^[14]^.

**Table 1:** Overview of tricuspid valve disease: classification, treatment options, devices, and complications.

**Classification of tricuspid valve disease**								
**Type**	**Etiology**	**Primary (%)**	**Secondary (%)**	**Mechanism**	**Common Causes**	**Severity Grading**	**Clinical Features**	**Diagnostic Modality**
Tricuspid Regurgitation (TR)	Organic/Functional	30	70	Incomplete leaflet coaptation	Rheumatic disease, prolapse, or annular dilation	Mild, Moderate, Severe	Fatigue, edema, ascites	Echocardiography (TTE/TEE), MRI
Tricuspid Stenosis (TS)	Organic	90	10	Restricted leaflet opening	Rheumatic disease, carcinoid syndrome	Mild, Moderate, Severe	Fatigue, right atrial enlargement	Echocardiography, CT
Mixed TR and TS	Combined	50	50	Combination of above mechanisms	Congenital, rheumatic disease	Mild, Moderate, Severe	Mixed symptoms	Echocardiography, 3D imaging
Congenital TVD	Structural	100	0	Congenital malformations	Ebstein anomaly, leaflet dysplasia	Varies	Cyanosis, heart failure in neonates	Fetal echocardiography, MRI
Functional TR	Secondary	0	100	Annular dilation, tethering	Left-sided heart failure, pulmonary HTN	Mild, Moderate, Severe	Dyspnea, systemic venous congestion	Echocardiography, catheterization
Isolated TR	Primary	100	0	Isolated leaflet involvement	Endocarditis, trauma	Mild, Moderate, Severe	Fever, murmur	Blood cultures, echocardiography
**Comparison of Treatment Modalities for Tricuspid Valve Disease**								
**Treatment Modality**	**Indications**	**Mechanism**	**Benefits**	**Limitations**	**Long-term Outcomes**	**Complications**	**Recovery Time**	**Cost Implications**
Medical Management	Early or mild TR/TS, symptomatic control	Diuretics, vasodilators	Non-invasive, cost-effective	Symptom-focused, no structural correction	Limited impact on disease progression	Fluid/electrolyte imbalances	Immediate relief	Low
Surgical Repair	Severe TR/TS with structural abnormalities	Annuloplasty, leaflet repair	Preserves native valve, effective	Requires open-heart surgery	Good long-term durability	Infection, arrhythmias	4–8 weeks	High
Surgical Replacement	Advanced or irreparable TR/TS	Valve replacement (bioprosthetic/mechanical)	Restores valve function, durable	High risk in complex cases	Dependent on valve type (bioprosthetic: ~ 10–15 yrs; mechanical: lifelong)	Thrombosis, prosthesis failure	4–8 weeks	Very high
Percutaneous Interventions	Moderate-severe TR in high-risk surgical patients	Catheter-based procedures (e.g., edge-to-edge repair, valve-in-valve)	Minimally invasive, shorter recovery	Limited availability, requires expertise	Promising outcomes (~70% symptom improvement)	Device dislodgement, vascular injury	1–2 weeks	Moderate to high
**Key Percutaneous Devices for Tricuspid Valve Interventions**								
**Device Name**	**Manufacturer**	**Mechanism of Action**	**Indications**	**Advantages**	**Limitations**	**Status**	**Clinical Trials Supporting the Device**	**Year of Development**
TriClip	Abbott	Edge-to-edge leaflet repair	Severe functional TR, high surgical risk	Minimally invasive	Limited to suitable anatomies	FDA-approved, CE-marked	TRILUMINATE Pivotal Trial	2020
Forma Repair System	Edwards Lifesciences	Spacer placed between leaflets to reduce regurgitation	Functional TR, moderate to severe	Simplified approach	Requires further trial validation	Investigational	Early feasibility trials	2018
Evoque	Edwards Lifesciences	Transcatheter valve replacement (TTVR)	Severe TR, degenerated bioprosthetic valves	Durable replacement	Risk of valve migration	Investigational	TRISCEND Study	2021
Cardioband Tricuspid System	Edwards Lifesciences	Annuloplasty via transcatheter approach	Severe functional TR, annular dilation	Adjustability of annular size	Technically complex	CE-marked	TRI-REPAIR Trial	2019
Navigate	Navigate Valve Inc.	Transcatheter valve implantation	Functional or primary TR	Adaptable for various anatomies	Limited clinical data	Investigational	NAVIGATE feasibility study	2022
**Procedural Complications and Management Strategies**								
**Complication**	**Incidence (%)**	**Risk Factors**	**Mechanism**	**Detection Method**	**Immediate Management**	**Long-term Management**	**Prevention Strategies**	**Prognosis**
Device Dislodgement	3–5	Suboptimal anchoring, annular calcification	Improper deployment	Echocardiography, fluoroscopy	Repositioning or device retrieval	Monitoring for residual TR	Improved operator training	Good if promptly managed
Vascular Injury	2–6	Large delivery systems, arterial calcification	Trauma during device delivery	Hemodynamic instability	Endovascular repair, surgical repair	Avoidance of large sheaths	Use of imaging guidance	Varies; better with prompt care
Right Ventricular Perforation	1–3	Device interaction with thin RV wall	Excessive force during intervention	Echocardiography	Pericardiocentesis, surgical repair	Monitoring for effusion	Careful manipulation of devices	Guarded if severe
Residual Tricuspid Regurgitation	10–15	Severe leaflet tethering, large annular dilation	Incomplete coaptation of leaflets	Echocardiography	Repeat intervention if symptomatic	Symptom management, diuretics	Optimal patient selection	Variable, impacts QoL
Infective Endocarditis	<1	Pre-existing infection, poor aseptic technique	Infection on device surfaces	Blood cultures, imaging	Antibiotics, device removal if needed	Long-term antibiotic therapy	Strict aseptic measures	Poor if untreated
Device Thrombosis	2–4	Hypercoagulable states, poor anticoagulation	Thrombus formation on prosthetic material	Echocardiography, CT	Anticoagulation therapy	Lifelong anticoagulation	Adequate anticoagulation protocols	Good with early detection
Heart Block	1–3	Close proximity to conduction system	Conduction system injury	ECG monitoring	Temporary or permanent pacemaker	Monitoring and device adjustment	Device positioning precautions	Good with pacing support
Hemorrhage	5–8	Anticoagulation therapy, vascular injury	Bleeding from access site or RV perforation	Monitoring for bleeding	Blood transfusion, surgical control	Avoidance of excessive anticoagulation	Careful sheath removal	Varies; depends on severity

Summarizes disease types, diagnostic approaches, treatment modalities, key devices, and associated complications. Source: Authors’ Creations.

**Table 2:** Comparison of imaging modalities for tricuspid valve disease.

Imaging Modality	Strengths	Limitations	Mechanism of Imaging	Diagnostic Accuracy	Interventional Utility	Availability	Cost Implications	Role in Diagnosis/Intervention
Transthoracic Echocardiography (TTE)	Non-invasive, widely available, low cost	Limited by poor acoustic windows in some patients	Uses sound waves to create images of the heart	High for initial assessment of TR	Limited for procedural guidance	Widely available	Low	Initial evaluation, assessment of TR severity
Transesophageal Echocardiography (TEE)	Superior image quality, good visualization of leaflets and annulus	Invasive, requires sedation or anesthesia	Sound waves via an esophageal probe	High for leaflet assessment	Excellent for guiding interventions	Available in specialized centers	Moderate	Detailed leaflet evaluation, procedural guidance
Computed Tomography (CT)	High spatial resolution, detailed anatomy visualization	Radiation exposure, contrast agent risks	X-ray-based imaging with cross-sectional views	Excellent for anatomical assessment	Limited real-time procedural use	Widely available	Moderate to high	Pre-procedure planning, annular anatomy assessment
Magnetic Resonance Imaging (MRI)	No radiation, excellent for functional assessment	Limited availability, contraindications in pacemaker patients	Magnetic fields for soft tissue imaging	High for functional TR analysis	Limited real-time procedural use	Limited to advanced centers	High	Functional assessment, pre-procedure evaluation
Fluoroscopy	Real-time imaging during procedures	Radiation exposure, lacks soft tissue detail	X-ray for dynamic imaging during interventions	Poor for diagnosis	Essential for device placement	Widely available	Moderate	Interventional device positioning, guidance
3D Echocardiography	Excellent spatial resolution, dynamic assessment	Operator-dependent, requires advanced equipment	3D reconstruction of sound wave data	Very high for anatomical details	Useful for complex procedural guidance	Limited to advanced centers	High	Complex anatomy visualization, procedural planning

Each modality has specific strengths: echocardiography for assessment and guidance, MRI for function, CT for anatomy, and fluoroscopy for interventions. Source: Authors’ Creations.

Secondary TR, on the other hand, is far more prevalent and results from functional impairment of the tricuspid valve despite structurally normal leaflets. This condition is often caused by right ventricular (RV) dilation or dysfunction, leading to annular dilation and tethering of the tricuspid leaflets. Pulmonary hypertension, left-sided heart failure, and atrial fibrillation are common etiologies of secondary TR. Chronic RV volume overload, as seen in conditions such as atrial septal defects or chronic pulmonary thromboembolism, further exacerbates the condition^[15]^. Importantly, secondary TR is often a late manifestation of other cardiac diseases, underscoring its complex pathophysiology and the interplay between right-sided and left-sided heart function.

The clinical manifestations of TR depend on its severity and underlying etiology. Mild cases may be asymptomatic and detected incidentally during echocardiography. In moderate to severe cases, patients often present with symptoms of right-sided heart failure, including fatigue, peripheral edema, ascites, and hepatic congestion. A holosystolic murmur at the lower left sternal border and signs of elevated jugular venous pressure may be evident on physical examination. The severity of TR is typically assessed using echocardiography, which provides information about valve anatomy, regurgitant volume, and RV function^[16]^.

On the other hand, TS, marked by restricted valve opening, impairs right ventricular filling and reduces cardiac output, often manifesting as fatigue and hepatomegaly^[12,13]^. The most frequent cause of TS worldwide is rheumatic heart disease, which leads to fibrotic thickening and calcification of the tricuspid leaflets, resulting in restricted leaflet mobility and narrowing of the valve orifice. In regions where rheumatic fever remains prevalent, TS is often seen in conjunction with mitral and aortic valve involvement, reflecting the systemic nature of the disease^[17]^.

Non-rheumatic causes of TS include congenital abnormalities, such as tricuspid atresia or unicuspid valves, and carcinoid heart disease, where serotonin-mediated plaque formation affects the tricuspid valve. Rarely, TS may result from infective endocarditis, often in intravenous drug users, or from mechanical obstruction due to a thrombus or tumor. Iatrogenic TS can occur in patients with pacemakers or defibrillator leads traversing the tricuspid valve, which may cause leaflet impingement or fibrosis^[18]^.

The pathophysiology of TS involves progressive obstruction of blood flow, leading to right atrial dilation, systemic venous congestion, and reduced RV filling. Symptoms typically develop insidiously, including fatigue, abdominal discomfort, and peripheral edema. On examination, a diastolic murmur is often heard along the lower left sternal border, which may be accentuated by maneuvers that increase venous return, such as inspiration.

Echocardiographic assessment is critical for diagnosis, with findings including thickened, immobile leaflets, reduced valve area, and elevated transvalvular pressure gradients^[19]^. Some patients may present with combined TR and TS, particularly in the context of rheumatic heart disease or carcinoid syndrome. The coexistence of stenosis and regurgitation poses unique challenges as the hemodynamic burden on the right atrium and ventricle is compounded. Management often requires addressing both lesions, with surgical or percutaneous interventions tailored to the underlying pathology and patient characteristics^[20]^.

One significant challenge in diagnosing TVD is the nonspecific nature of its clinical manifestations. Mild to moderate TR is often asymptomatic and may remain undetected for years, particularly in patients with no prior history of heart disease. When symptoms do arise, they tend to overlap with those of other conditions, such as heart failure or pulmonary hypertension, leading to delayed or misdiagnosis. Common symptoms like fatigue, peripheral edema, ascites, and dyspnea are not unique to TVD and may be attributed to left-sided heart failure or systemic diseases. This diagnostic ambiguity is particularly problematic in secondary TR, where the disease is often a sequela of left-sided heart failure, atrial fibrillation, or pulmonary hypertension, diverting attention from the tricuspid valve itself^[21-23]^.

Another challenge lies in the limitations of physical examination in detecting TVD. While murmurs associated with TVD, such as the holosystolic murmur of TR or the diastolic murmur of TS, may be detected on auscultation, their sensitivity and specificity are limited. These murmurs are often soft or inaudible, especially in obese or critically ill patients, and their detection requires skilled clinicians and optimal conditions. Furthermore, the signs of right-sided heart failure, such as jugular venous distension or hepatomegaly, are not specific to TVD and can be missed in early stages or masked by coexisting conditions.

**Figure 1.**
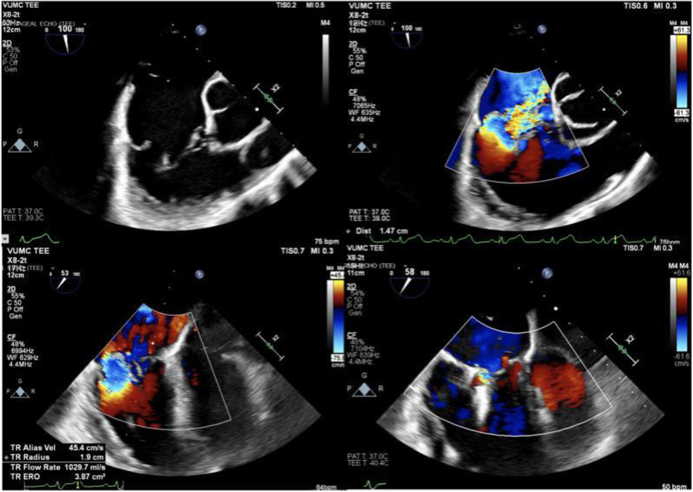
Transesophageal echocardiography showing TriClip tricuspid valve repair. Transesophageal echocardiography image demonstrating TriClip tricuspid valve repair. Tricuspid regurgitation improved from torrential to trace after placement of an anterior-posterior leaflet device and a second device in the posterior-septal location. Source: Authors’ Creations.

Echocardiography, the cornerstone of TVD diagnosis, also poses diagnostic challenges. While transthoracic echocardiography (TTE) is widely available and non-invasive, it may provide suboptimal imaging of the tricuspid valve due to its posterior location in the heart^[24,25]^. Acoustic windows are often limited, particularly in patients with obesity or chronic lung disease, making it difficult to visualize the valve leaflets, annulus, and subvalvular apparatus. Transesophageal echocardiography (TEE) offers improved imaging quality but is more invasive and not routinely performed, which limits its accessibility (see Fig. 1).

Additionally, accurately quantifying the severity of TR or TS can be challenging, as current grading systems rely on multiple echocardiographic parameters that may be operator-dependent and influenced by loading conditions. Variability in measurement techniques and interpretation further complicates the assessment, leading to inconsistencies in diagnosis and classification^[26,27]^. Advanced imaging modalities, such as cardiac magnetic resonance (CMR) and three-dimensional echocardiography, provide valuable insights into the anatomy and function of the tricuspid valve and the right ventricle. However, these modalities are not universally available, requiring specialized expertise and equipment. CMR, for instance, is particularly useful for assessing RV volumes, function, and regurgitant fraction, but its high cost and limited availability in resource-constrained settings restrict its widespread use. Similarly, three-dimensional echocardiography enhances the visualization of tricuspid valve morphology and dynamics but is not yet a routine part of clinical practice^[28,29]^.

A further diagnostic challenge is the underrepresentation of TVD in clinical guidelines and awareness among clinicians. Historically, the tricuspid valve has been regarded as the “forgotten valve,” receiving less attention than the mitral and aortic valves in research, clinical practice, and education. This has led to a lack of standardized diagnostic criteria and protocols for TVD, resulting in variations in practice and potential underdiagnosis. Moreover, TVD often occurs in older patients or those with multiple comorbidities, where the focus may be on managing more apparent or symptomatic conditions, inadvertently overlooking tricuspid valve pathology^[30,31]^.

Invasive hemodynamic assessment, such as right heart catheterization, is sometimes required for definitive diagnosis, particularly in TS or severe TR cases with ambiguous non-invasive findings. However, this procedure is invasive, associated with risks, and not always feasible in critically ill patients. Additionally, the interpretation of hemodynamic data can be complex, especially in patients with mixed valve disease or significant pulmonary hypertension^[32-34]^.

## Traditional management strategies

Tricuspid valve disease (TVD), including regurgitation (TR) and stenosis (TS), leads to systemic venous congestion and progressive right heart failure^[20]^. Medical therapy primarily offers symptomatic relief, especially in patients ineligible for surgery or percutaneous procedures. Diuretics, particularly loop agents like furosemide, are central in managing volume overload, with adjunctive use of thiazides or aldosterone antagonists like spironolactone in resistant cases (see Table 1) ^[21,22]^. Monitoring for electrolyte imbalances and renal function is essential during diuretic therapy^[23]^.

Pulmonary vasodilators, such as sildenafil and bosentan, may reduce TR severity in patients with pulmonary hypertension^[24]^. ACE inhibitors or ARBs can be helpful in coexistent left-sided heart disease, though their role in isolated TR remains limited^[25]^. Rate control in atrial fibrillation, common in advanced TVD, is achieved with beta-blockers or calcium channel blockers, while amiodarone may be considered for rhythm control^[26,27]^. Anticoagulation with DOACs or VKAs is indicated when atrial fibrillation coexists^[28]^. Inotropic support, such as dobutamine or milrinone, is reserved for decompensated right heart failure with low cardiac output^[29]^.

Managing secondary TR involves treating underlying causes, such as optimizing left ventricular function with guideline-directed therapy (e.g., beta-blockers, ACEIs, MRAs) and controlling pulmonary hypertension^[30]^. However, current therapies target symptoms rather than disease modification. Novel pharmacologic agents and personalized approaches, such as genetic and biomarker-based strategies, are under investigation^[31]^. The lack of large-scale clinical trials further limits the evidence-based optimization of medical therapy in TVD^[32]^.

Surgical intervention remains the mainstay for severe symptomatic TR or TS unresponsive to medical therapy, especially during concurrent left-sided valve surgery (see Table 4) ^[11,21]^. Valve repair – particularly with annuloplasty – is preferred, though replacement with bioprosthetic or mechanical valves is necessary in advanced cases^[14,15]^. Surgical timing is crucial; delayed intervention in severe TR often leads to irreversible RV dysfunction and worse outcomes^[16]^. High perioperative risks and under-recognition of TVD contribute to surgical underutilization, a challenge referred to as the “forgotten valve” phenomenon^[17,18]^.

Minimally invasive and robotic-assisted techniques are emerging to reduce surgical morbidity^[19]^. Despite progress, significant unmet needs persist in TVD management, highlighting the need for earlier diagnosis, better medical therapies, and optimized intervention timing^[20-26]^.

## Emergence of percutaneous interventions

The historical perspective of percutaneous cardiac interventions begins with their application in coronary artery disease. The introduction of balloon angioplasty by Andreas Grüntzig in 1977 marked the inception of minimally invasive procedures in cardiology. This milestone was followed by stenting, which further revolutionized revascularization.

Building on these foundational advancements, percutaneous techniques expanded into structural heart disease with the development of TAVR in the early 2000s. TAVR provided a less invasive option for high-risk patients with aortic stenosis, demonstrating the potential for transcatheter approaches to address complex valvular pathology^[33]^. Success in the aortic and mitral spaces provided the impetus for exploring similar interventions in the tricuspid position. The tricuspid valve, anatomically and physiologically distinct from other cardiac valves, presented unique challenges, necessitating device-specific innovations to accommodate its lower-pressure system, asymmetrical annulus, and proximity to critical structures such as the right coronary artery and conduction pathways^[34]^.

The rationale for percutaneous approaches to TVD stems from the limitations of existing treatment paradigms. Medical management, often centered on diuretics, addresses symptoms of volume overload but does not reverse disease progression or mitigate structural deterioration. On the other hand, surgical repair or replacement, while effective, carries significant risks, particularly in patients with advanced age, comorbidities, or previous cardiac surgeries. These considerations are particularly relevant given the epidemiological profile of TR, which predominantly affects elderly patients with multiple systemic conditions. Data from large registries suggest that up to 50% of patients with significant TR are deemed ineligible for surgery due to prohibitive risk profiles, leaving a substantial unmet clinical need^[35]^.

**Figure 2.**
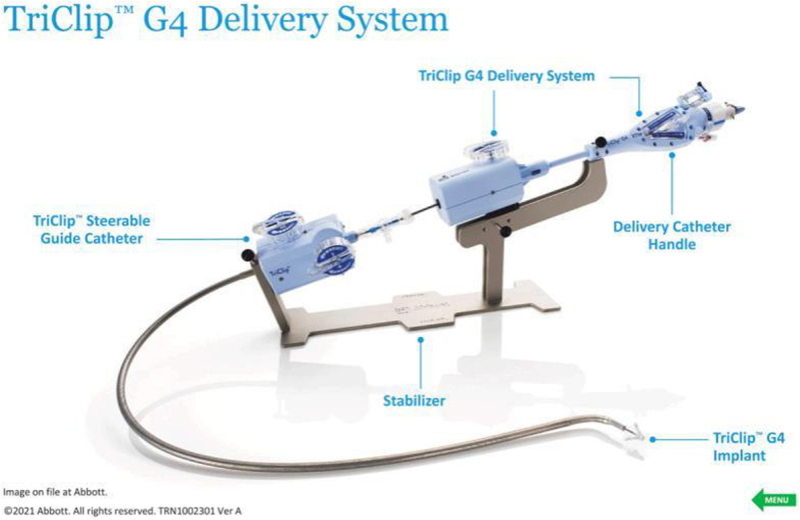
The Abbott TriClip G4 delivery system and edge-to-edge repair device. The Abbott TriClip G4 delivery system and edge-to-edge repair device designed for minimally invasive tricuspid valve repair. Source: Authors’ Creations.

**Figure 3.**
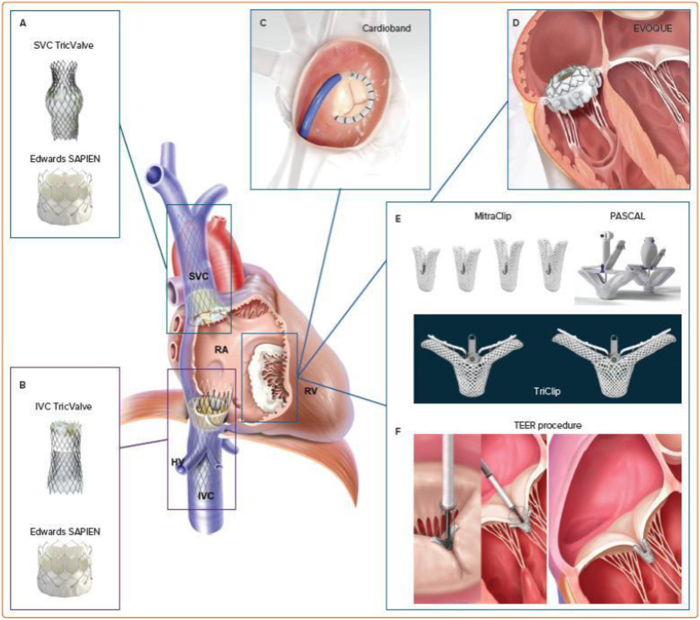
Devices for percutaneous tricuspid regurgitation therapy, classified by anatomical locations. Various devices are categorized based on their application to anatomical regions of the tricuspid valve, including leaflet repair, annular modification, and caval approaches. Source: Authors’ Creations.

Percutaneous interventions, by contrast, offer the promise of targeted therapy with reduced procedural morbidity. Techniques such as edge-to-edge repair, modeled after the success of the MitraClip in mitral regurgitation, have demonstrated feasibility and safety in addressing tricuspid pathology. For instance, the TriClip system, specifically designed for tricuspid valve repair, has shown encouraging results in reducing TR severity and improving functional status in high-risk patients (see Fig. 2)^[36]^. Similarly, annuloplasty devices, which reshape and stabilize the tricuspid annulus, provide an alternative mechanism for addressing regurgitation (see Fig. 3). Early experiences with devices such as the Cardioband and Trialign systems suggest that percutaneous annuloplasty can achieve meaningful reductions in annular dimensions and regurgitant volume, translating to symptomatic relief^[37]^.

The benefits of minimally invasive procedures over traditional surgery are multifaceted, encompassing clinical, logistical, and economic domains. Clinically, percutaneous approaches reduce the need for sternotomy, cardiopulmonary bypass, and prolonged general anesthesia, all of which are associated with higher perioperative risk in frail patients. Furthermore, these interventions are often performed via venous access, eliminating the need for arterial puncture and reducing the likelihood of vascular complications. Postoperative recovery is expedited, with shorter hospital stays and quicker return to baseline functional status^[9]^. These advantages are particularly pronounced in older adults, where surgical trauma can precipitate complications such as frailty exacerbation, delirium, and prolonged rehabilitation.

Logistically, the scalability of percutaneous interventions is a significant advantage, allowing more widespread adoption in settings with varying resource availability. Unlike surgery, which often requires specialized cardiac surgical teams and facilities, many percutaneous procedures can be performed in catheterization laboratories equipped with fluoroscopic and echocardiographic imaging capabilities. This reduces the infrastructural burden and facilitates broader access to advanced therapies, particularly in underserved regions.

Economically, percutaneous interventions may offer cost savings by reducing hospitalization duration, minimizing postoperative complications, and potentially delaying the need for subsequent interventions (see Table 3). While the upfront costs of devices and procedural expertise are substantial, downstream savings in healthcare utilization may offset these expenses. Although limited at this stage, cost-effectiveness analyses suggest that percutaneous approaches are likely economically viable in high-risk populations, particularly when long-term outcomes such as improved survival and quality of life are considered^[10,11]^.

**Table 3:** Economic and health system considerations of percutaneous interventions for tricuspid valve disease.

Parameter	Cost Implications	Hospital Stay/Recovery Time	Impact on Healthcare Resource Utilization	Cost-Effectiveness Analysis (if available)	Procedure Duration	Long-Term Follow-Up Requirements	Impact on Patient Quality of Life	Overall Economic Burden
Percutaneous Device Costs	High upfront costs (devices range $20k–$50k)	Shorter stay (1–3 days)	Reduced burden compared to surgical interventions	Studies suggest cost-effectiveness in high-risk patients	1–2 hours	Regular imaging follow-ups required	Improved due to faster recovery	Moderate to high
Surgical Alternatives (Replacement)	Lower initial cost compared to devices	Longer stay (5–7 days)	High ICU and resource utilization post-op	Less cost-effective in elderly/high-risk groups	4–6 hours	Lifelong anticoagulation monitoring	Delayed improvement due to longer recovery	High
Hospital Infrastructure Costs	Requires specialized cath lab setup	Cath labs reduce hospital ward usage	Frees up ICU beds for other patients	Limited data on system-wide cost implications	Moderate to low	Minimal, compared to surgeries	Reduced hospitalization stress	Moderate
Medication Costs Post-Procedure	Lower anticoagulant use in selected devices	Minimal medication requirements	Reduces long-term pharmacological burden	Favorable in terms of reducing medication load	N/A	Anticoagulants for specific devices	Positive, due to reduced medical dependency	Low
Readmission Rates	Lower compared to surgical procedures	Faster recovery reduces readmission risk	Decreases long-term hospital resource use	Cost-effective in reducing overall readmission	N/A	Lower readmission requirements	Positive outcomes enhance well-being	Low
Physician Training Requirements	High initial training costs	N/A	Increased demand for trained specialists	Yet to be evaluated in long-term analyses	N/A	Required for newer technologies	Indirect impact through better outcomes	High
Health System Efficiency	High procedural efficiency offsets costs	Faster patient turnover	Improved allocation of hospital resources	Indirect cost benefits noted	Short	Lower burden on long-term care systems	High impact through efficiency gains	Moderate
Patient Outcomes (QoL Gains)	High investment results in superior outcomes	Short recovery time	Improved quality of care metrics	Strong cost-benefit alignment	N/A	Minimal long-term management required	Substantial improvement noted	Positive

Key parameters include costs, hospital stay, resource use, quality of life, and overall system impact. Source: Authors’ Creations.

**Table 4:** Guidelines for percutaneous interventions in tricuspid valve disease.

Guideline/Organization	Key Recommendations	Patient Selection Criteria	Evidence Supporting Recommendations	Recommended Devices	Strength of Evidence	Applicability Across Regions	Follow-Up Requirements	Impact on Clinical Outcomes
ACC/AHA (American College of Cardiology/American Heart Association)	Use percutaneous interventions for patients with severe TR unresponsive to medical therapy	Patients with high surgical risk, symptomatic severe TR, or annular dilation	Based on pivotal trials (e.g., TRILUMINATE)	TriClip, Forma Repair System	Class IIa, Level of Evidence B	High in high-resource settings	Regular imaging and clinical follow-up	Improved symptom relief, quality of life
ESC (European Society of Cardiology)	Percutaneous treatment recommended for functional TR secondary to left-sided heart disease	Patients with functional TR and evidence of right ventricular overload or annular dilation	Supported by registries and small clinical trials	Cardioband, TriClip	Class IIa, Level of Evidence C	Variable, depending on device availability	Annual imaging required	Reduction in heart failure symptoms
EACTS (European Association for Cardio-Thoracic Surgery)	Percutaneous repair preferred for elderly and frail patients not eligible for surgery	High-risk patients with anatomical suitability for device implantation	Early clinical evidence from multicenter studies	TriClip, Pascal	Class IIb, Level of Evidence C	Limited in low-resource settings	Device-specific imaging protocols	Reduced hospitalizations, improved QoL
NICE (National Institute for Health and Care Excellence, UK)	Evaluate percutaneous tricuspid interventions for specific patient cohorts in clinical trials	Patients with severe TR ineligible for surgical repair and meeting anatomical criteria	Limited evidence; recommendations based on emerging clinical data	Investigational Devices	Not rated yet	Primarily in clinical trial settings	Trial-specific follow-up requirements	Yet to be determined
Canadian Cardiovascular Society (CCS)	Percutaneous options should be explored before surgical approaches in high-risk patients	Patients with symptomatic TR or progressive RV dysfunction despite optimized medical management	Supported by ongoing studies like TRILUMINATE-CE	TriClip, Trialign	Class IIa, Level of Evidence B	Available in major cardiac centers	Lifelong follow-up, imaging every 6 months	Significant reduction in hospital visits
Asia-Pacific Society of Cardiology (APSC)	Encourages research and adoption of cost-effective percutaneous interventions	Patients with limited access to surgery but meeting criteria for device-based therapies	Emerging studies and registries	Limited options currently	Class IIb, Level of Evidence C	Low to moderate, depending on infrastructure	Annual clinical follow-up	Positive in select patient populations
World Health Organization (WHO)	Advocates equity in access to emerging cardiovascular interventions	Resource-poor settings with appropriate patient selection criteria	Limited global evidence; focuses on pilot programs	Investigational and approved devices	N/A	Limited in low-income regions	N/A	Requires further research
Japanese Circulation Society (JCS)	Focuses on anatomical suitability and advanced imaging guidance for interventions	Patients with severe symptomatic TR, particularly in the context of pacemaker-induced regurgitation	Evidence from Japanese registries and localized trials	Cardioband, TriClip	Class IIa, Level of Evidence C	High in developed settings	Device-specific follow-up protocols	Improved patient satisfaction
American Society of Echocardiography (ASE)	Recommends integrating advanced imaging for patient selection and procedural guidance	Patients with moderate to severe TR and suitable annular dimensions for device implantation	High-resolution echocardiographic evidence; supports precision in device placement	Advanced imaging modalities	Class I, Level of Evidence B	High across advanced centers	Standard echocardiographic follow-up	Positive procedural outcomes

Summarizes key recommendations from major societies on patient selection, imaging, device use, follow-up, and expected outcomes. Source: Authors’ Creations.

Despite these advancements, challenges remain. Procedural success is contingent on precise imaging, device positioning, and operator expertise, necessitating ongoing training and technological refinements. Long-term durability data for many devices are currently lacking, underscoring the need for robust post-market surveillance and clinical trials. Nevertheless, the paradigm shift represented by percutaneous interventions in TVD is undeniable. By addressing a historically neglected valve with innovative, patient-centered solutions, these techniques have redefined the therapeutic landscape, offering hope to a previously underserved population.

## Types of percutaneous interventions for tricuspid valve disease

Percutaneous interventions for TVD have evolved significantly, offering new therapeutic avenues for patients who are often deemed high-risk for traditional surgical approaches. This paradigm shift addresses the growing recognition of the tricuspid valve’s role in cardiac health and the challenges posed by TVD, particularly TR^[38]^. These interventions can broadly be categorized into tricuspid valve repair and replacement, each with distinct mechanisms, indications, and clinical implications.

Tricuspid valve repair strategies, particularly edge-to-edge repair, have gained prominence due to their minimally invasive nature and favorable outcomes in reducing regurgitation severity. Devices such as the MitraClip, initially designed for the mitral valve, have been adapted for tricuspid valve applications. The TriClip system, for example, is a modification specifically targeting tricuspid valve leaflets (see Table 1). Edge-to-edge repair involves approximating the tricuspid valve leaflets, thereby reducing regurgitation by improving leaflet coaptation. This approach is particularly beneficial in patients with functional TR secondary to right ventricular dilatation or annular enlargement^[39-41]^.

Early studies, such as the TRILUMINATE trial, have demonstrated promising results, with significant reductions in regurgitation severity and improved functional status and quality of life among treated patients^[1,2]^. The edge-to-edge technique is relatively straightforward and can be performed under echocardiographic and fluoroscopic guidance, making it an attractive option for high-risk patients.

Annuloplasty devices represent another critical innovation in tricuspid valve repair. These devices aim to restore the anatomical shape and function of the tricuspid valve annulus, which is often dilated in cases of functional TR. Percutaneous annuloplasty systems, such as the Cardioband Tricuspid System and the Trialign device, utilize transcatheter techniques to reshape the annulus and reduce the regurgitant orifice area.

Cardioband, for example, involves implanting a flexible band around the annulus via a transvenous approach, with subsequent tensioning to achieve annular reduction. Similarly, the Trialign device employs suture-based plication to approximate the annulus, effectively reducing its diameter. Clinical trials evaluating these devices, including the SCOUT trial for Cardioband, have demonstrated encouraging results regarding safety, feasibility, and reductions in TR severity^[3,4]^. Annuloplasty devices are particularly suitable for patients with significant annular dilation but relatively preserved leaflet structure and function.

Tricuspid valve replacement, on the other hand, represents an entirely different therapeutic approach. Unlike repair techniques, replacement involves the implantation of a new valve, either within the native valve or in previously implanted prosthetic valves or rings. Transcatheter tricuspid valve replacement (TTVR) has emerged as a viable option for patients with severe TR who are not candidates for repair.

Devices such as the EVOQUE and GATE valves are specifically designed for TTVR. The EVOQUE valve, for instance, is a self-expanding, bioprosthetic valve delivered via a transjugular approach. It anchors securely within the native tricuspid valve annulus, immediately eliminating regurgitation. Early clinical experiences with the EVOQUE valve have shown high procedural success rates and marked improvements in clinical outcomes, as evidenced in studies like the TRICENTRIC trial^[42,43]^.

Valve-in-valve and valve-in-ring procedures are innovative adaptations of transcatheter techniques for treating degenerated bioprosthetic valves or failed annuloplasty rings in the tricuspid position. These procedures involve implanting a new valve within the prosthetic structure, effectively restoring valve function without opening-heart surgery. The Sapien XT and Sapien 3 valves, initially developed for aortic applications, are commonly used in these procedures. Their deployment in the tricuspid position is guided by advanced imaging modalities, ensuring accurate placement and optimal outcomes (see Table 2). Studies have demonstrated the feasibility and safety of these approaches, with favorable short-term and mid-term results in reducing regurgitation and improving hemodynamics^[44,45]^.

Valve-in-valve and valve-in-ring procedures are particularly valuable for patients with prior surgical interventions in the tricuspid position, offering a less invasive option for managing prosthetic valve dysfunction.

Emerging devices and technologies continue to expand the landscape of percutaneous interventions for TVD. Innovations in imaging, navigation systems, and device design address the technical challenges associated with percutaneous tricuspid interventions. The TriAlign and Forma Repair systems represent the next frontier in tricuspid valve repair. The Forma device, for instance, utilizes a spacer positioned in the regurgitant orifice to reduce the effective regurgitant area, thereby improving valve function.

Additionally, novel replacement devices, including the LuX-Valve and the NaviGate system, are being developed to enhance procedural efficiency and durability. Ongoing clinical trials and registries evaluate these emerging technologies, providing critical insights into their safety, efficacy, and long-term outcomes^[24,46]^.

## Comparative analysis with left-sided valve interventions

Anatomically, the tricuspid valve differs significantly from the mitral and aortic valves. The TV is a three-leaflet structure anchored by chordae tendineae and papillary muscles, similar to the mitral valve but structurally more delicate. The leaflets are thinner and more mobile, and the annulus is elliptical and dynamic, expanding and contracting with the phases of the cardiac cycle. The tricuspid annulus is more susceptible to dilation due to its proximity to the right atrium, which is prone to volume overload in conditions such as atrial fibrillation and pulmonary hypertension^[47,48]^. This contrasts with the relatively rigid mitral and aortic annuli.

Additionally, the proximity of the tricuspid valve to the conduction system, including the atrioventricular node, increases the risk of conduction disturbances during interventions. These anatomical features underscore the unique challenges of developing tricuspid valve repair and replacement devices.

From a pathophysiological standpoint, the primary disease mechanism in tricuspid valve pathology is often secondary TR, driven by right ventricular dilation and annular enlargement^[23]^. Unlike left-sided valve diseases, which are frequently driven by intrinsic leaflet abnormalities (e.g., mitral valve prolapse or aortic stenosis), tricuspid valve pathology is more often a consequence of systemic or pulmonary conditions. The lower-pressure right-sided circulation also means that TR may remain asymptomatic for longer, delaying diagnosis and treatment. This contrasts mitral and aortic valve diseases, where higher pressures lead to earlier symptom onset and intervention.

Despite these differences, the advancements in percutaneous mitral and aortic valve interventions have provided invaluable insights applicable to tricuspid valve therapies^[49-51]^. The development of edge-to-edge repair devices, such as the MitraClip, has laid the foundation for similar approaches in the tricuspid valve. Devices like the TriClip and PASCAL, which leverage principles from mitral valve repair, have shown promise in reducing TR severity in clinical trials.

Furthermore, the success of TAVR has underscored the importance of precise imaging and device delivery, principles that are now being applied to tricuspid valve replacement technologies, such as the EVOQUE and GATE systems. However, unique challenges specific to percutaneous tricuspid valve interventions remain. The low-pressure venous circulation of the right side poses difficulties in device anchoring and stability. Unlike the aortic valve, which benefits from calcified annuli that provide a stable landing zone for transcatheter valves, the tricuspid annulus often lacks significant calcification, necessitating innovative anchoring mechanisms^[21,52,53]^. Additionally, the larger leaflet area and dynamic nature of the tricuspid annulus make achieving coaptation more complex. The risk of device interference with adjacent structures, such as the right coronary artery or pacemaker leads, adds another layer of complexity.

Another critical challenge is the heterogeneity of TVD and patient anatomy, which complicates patient selection and procedural standardization. While mitral and aortic valve interventions have benefited from well-defined guidelines and patient cohorts, the tricuspid valve population is more diverse, with a wide range of anatomical and clinical presentations. This necessitates a more personalized approach to device selection and procedural planning^[54,55]^.

Despite these obstacles, the lessons learned from left-sided valve interventions provide a roadmap for overcoming these challenges. The integration of advanced imaging techniques, such as 3D echocardiography and CT, has been pivotal in mitral and aortic valve procedures and is now being adapted for tricuspid interventions. These imaging modalities enable detailed assessment of valve anatomy, annular dimensions, and the spatial relationship of adjacent structures, facilitating precise device placement and reducing the risk of complications^[15,56]^.

The multidisciplinary “Heart Team” approach, which has been instrumental in the success of TAVR and MitraClip procedures, is equally critical for tricuspid valve interventions. Collaboration among interventional cardiologists, cardiac surgeons, echocardiographers, and anesthesiologists ensures comprehensive patient evaluation and procedural planning. Moreover, the emphasis on patient-centered care, including considering quality-of-life metrics and patient preferences, aligns with the broader goals of modern cardiac care.

## Clinical evidence and outcomes

Clinical trials such as TRILUMINATE and the early feasibility studies on MitraClip for TR have provided robust evidence for the efficacy of percutaneous interventions. The TRILUMINATE trial specifically evaluated the TriClip device in patients with symptomatic, severe TR deemed high-risk for surgery. Results demonstrated a marked reduction in TR severity, with approximately 86% of patients achieving TR reduction to moderate or less at one year. Moreover, improvements were noted in functional capacity as assessed by the six-minute walk test (6MWT) and quality of life scores using the Kansas City Cardiomyopathy Questionnaire (KCCQ)^[1,2]^. These findings align with those observed in MitraClip trials for mitral regurgitation, highlighting the broader applicability of edge-to-edge repair techniques in valve disease management.

Real-world registries like the TriValve registry have also provided essential insights. This registry tracks outcomes of transcatheter tricuspid interventions across multiple centers worldwide, offering a comprehensive view of procedural success rates, safety, and patient-reported outcomes. Analysis of over 300 cases from the registry revealed a procedural success rate of 91%, defined as a reduction of TR to moderate or less without significant complications. Importantly, significant improvements were observed in the New York Heart Association (NYHA) functional class, with a shift from predominantly class III/IV pre-procedure to class I/II post-procedure in over 75% of patients^[3,4]^.

Safety profiles of percutaneous interventions have been favorable, with low rates of procedure-related complications. Device dislodgment, leaflet perforation, and right ventricular injury remain rare but notable risks. The TRILUMINATE trial reported a device-related complication rate of 1.7%, underscoring the procedural safety of modern percutaneous techniques^[2]^. Additionally, long-term follow-up data from early feasibility studies have demonstrated sustained reductions in TR severity, suggesting promising durability of these interventions.

Impact on quality of life and functional outcomes has been consistently demonstrated across studies. Patients undergoing percutaneous tricuspid valve repair report significant symptomatic relief, with marked reductions in edema, ascites, and other right-sided heart failure symptoms. The KCCQ scores, often used as a benchmark for quality of life in valve disease trials, have shown mean increases of 18–22 points post-intervention, exceeding the threshold for clinically meaningful improvement^[5,6]^. Furthermore, improvements in exercise tolerance, as reflected by 6MWT distances, have been consistently reported, with mean increases of 50–70 m at 6 months post-procedure^[1,4]^.

Survival outcomes, although less extensively studied, have been encouraging. A meta-analysis of percutaneous tricuspid valve interventions, encompassing data from over 1000 patients, demonstrated a significant reduction in 1-year mortality compared to conservatively managed cohorts. The hazard ratio for all-cause mortality was 0.65, favoring percutaneous approaches, even after adjusting for baseline comorbidities and TR severity^[7]^. These findings are particularly relevant given the high mortality associated with severe, untreated TR, which approaches 35% at one year in medically managed patients^[8]^.

TTVR is another promising area, with devices such as the EVOQUE and GATE valves undergoing rigorous evaluation. Early feasibility studies for TTVR have reported procedural success rates exceeding 90%, with sustained reductions in TR severity and improvements in right ventricular function at 6 months. Although long-term data are still emerging, preliminary results suggest favorable outcomes comparable to those of transcatheter aortic and mitral valve replacements^[9]^.

Despite these advances, challenges remain in patient selection and device optimization. Current trials are primarily limited to patients with functional TR secondary to left-sided heart disease or pulmonary hypertension. Structural abnormalities of the tricuspid valve, such as in Ebstein’s anomaly, present unique challenges that require further innovation. Additionally, the lack of robust randomized controlled trials (RCTs) limits the generalization of findings, particularly in diverse patient populations. Ongoing RCTs such as TRILUMINATE Pivotal and CLASP II TR are expected to address these gaps, providing high-quality evidence to inform clinical practice^[10,11]^.

Integrating imaging modalities, including 3D transesophageal echocardiography and cardiac MRI, has enhanced procedural planning and outcomes assessment. These tools allow for precise quantification of TR severity, right ventricular function, and device positioning, ensuring optimal outcomes. Advances in imaging-guided interventions, coupled with the development of next-generation devices, are anticipated to improve success rates further and expand percutaneous approaches’ applicability.

## Challenges and limitations

The rise of percutaneous interventions in TVD has significantly improved treatment options for patients who were previously deemed ineligible for surgery due to comorbidities and high surgical risk. However, these advancements are not without challenges and limitations. One of the primary challenges in adopting percutaneous interventions lies in patient selection and anatomical variability. Unlike mitral and aortic valves, the tricuspid valve presents unique anatomical and physiological complexities. Its thin, delicate leaflets and large annular dimensions, which often increase further in disease states, pose significant challenges for device anchoring and procedural success^[1]^.

Patients with TR often present secondary etiologies linked to right ventricular dilatation and annular enlargement, challenging uniform device deployment. Furthermore, extensive calcification, prior cardiac surgeries, or pacemaker leads complicate patient eligibility for these procedures^[2]^. Identifying candidates who will benefit most is difficult, as current guidelines lack definitive recommendations on percutaneous treatment indications, leaving room for subjective clinical judgment.

Technical limitations and complications during percutaneous procedures further complicate adopting these approaches. Tricuspid valve interventions require advanced imaging modalities, such as transesophageal echocardiography and cardiac computed tomography (CT), to visualize the intricate anatomy adequately. However, imaging of the right heart is inherently more complex due to interference from adjacent structures and the variable positioning of the valve. This limits procedural precision and increases the risk of suboptimal device placement^[3]^.

Device-specific challenges, such as achieving stable anchoring of annuloplasty rings or repair devices, can also lead to procedural failure. Moreover, navigating the right heart’s low-pressure system poses unique hemodynamic risks during interventions, potentially resulting in complications such as valve leaflet perforation, device embolization, or arrhythmias^[4,5]^. Other issues, such as TS following annuloplasty or residual regurgitation, can undermine procedural outcomes and necessitate repeat interventions.

The long-term durability of the implanted devices remains a significant concern, particularly given the recent advent of tricuspid valve interventions. While short- to mid-term data suggest encouraging results regarding symptom improvement and quality of life, robust evidence on long-term outcomes is scarce. This is partly because most trials evaluating percutaneous tricuspid devices have focused on feasibility and early outcomes rather than durability and survival^[6]^. Additionally, the high prevalence of comorbidities in this patient population complicates long-term follow-up, as competing risks of mortality may obscure device-related complications.

This lack of long-term data raises questions about device reliability and necessitates ongoing clinical surveillance through post-market registries and extended trials^[7]^. Issues such as thromboembolic events, device erosion, and late-onset infections further underscore the importance of monitoring outcomes over a longer horizon. These challenges underscore the need for a cautious yet proactive approach to implementing percutaneous interventions for TVD. A multidisciplinary strategy involving cardiologists, interventionalists, and imaging specialists is essential to optimize patient selection, refine procedural techniques, and establish robust long-term follow-up protocols. Addressing these limitations will be pivotal to ensuring the sustained success of these promising therapies in clinical practice.

## Future directions

The landscape of percutaneous interventions in TVD is evolving rapidly, with numerous promising advancements that aim to enhance procedural success, expand patient access, and optimize outcomes. One of the most significant areas of progress is the continuous innovation in device technology and procedural techniques. Current devices, such as the MitraClip adapted for the tricuspid valve, and novel devices like the TriClip, PASCAL system, and the EVOQUE transcatheter valve replacement system, represent pivotal advances in tricuspid valve repair and replacement^[1,2]^. These technologies are engineered to address the unique anatomical challenges the tricuspid valve poses, such as its larger size, asymmetrical leaflets, and proximity to critical structures like the conduction system.

Newer annuloplasty systems, such as the Cardioband Tricuspid and TriAlign systems, are refined to improve durability and procedural success^[3]^. Meanwhile, valve-in-valve and valve-in-ring procedures are gaining traction, especially in patients with prior surgical interventions, offering minimally invasive solutions for recurrent TR. Emerging technologies are focused on enhancing the precision and efficiency of these interventions. For instance, steerable catheters and delivery systems enable more accurate device placement, reducing procedural time and complications^[4]^. Furthermore, next-generation devices integrate real-time feedback mechanisms that allow operators to adjust during the procedure, improving outcomes. Continued innovation in materials science is also pivotal, with biocompatible and thromboresistant materials reducing the risk of device-related complications such as thrombosis and endocarditis^[5]^.

Expanding indications and patient eligibility is crucial in ensuring that more patients benefit from percutaneous interventions. Historically, patients with significant comorbidities or advanced age were often deemed unsuitable for surgical repair or replacement due to the high perioperative risk^[6]^. However, minimally invasive techniques have shifted this paradigm, making treating high-risk patients with fewer complications and shorter recovery times possible. As device technology advances, indications for percutaneous interventions will likely broaden to include patients with moderate TR who are symptomatic but not yet candidates for surgery^[7]^. Additionally, early intervention strategies are being explored, with the hypothesis that addressing TR before the onset of severe right ventricular dysfunction could prevent long-term morbidity and mortality.

Pediatric and congenital heart disease populations also stand to benefit from these advancements. Percutaneous interventions are being tailored to address congenital anomalies affecting the tricuspid valve, such as Ebstein’s anomaly, where traditional surgical options may be limited^[8]^. Ongoing efforts to miniaturize devices and optimize delivery systems for smaller anatomical structures will further expand the applicability of these interventions to younger patients.

Despite these promising developments, there is an urgent need for robust clinical trials and registries to validate the safety and efficacy of percutaneous tricuspid valve interventions. Most existing studies are observational or involve small patient cohorts, limiting the generalizability of their findings^[9]^. Large-scale, randomized controlled trials (RCTs) are necessary to establish standardized guidelines and protocols for patient selection, procedural techniques, and post-procedure management. Registries like the TRILUMINATE trial are pivotal in collecting real-world data on device performance, long-term outcomes, and complication rates^[10]^. These initiatives are essential for addressing knowledge gaps and informing clinical practice.

Longitudinal studies are also critical for evaluating the durability of percutaneous devices over time. Unlike surgical repairs, which have well-documented long-term outcomes, percutaneous interventions are relatively new, and their durability remains a subject of ongoing investigation^[11]^. Data from multi-center registries and post-market surveillance programs will provide valuable insights into device longevity, the risk of structural deterioration, and the need for reintervention.

Integrating artificial intelligence (AI) and advanced imaging modalities is poised to revolutionize the planning and execution of percutaneous tricuspid valve interventions. AI algorithms are being developed to analyze patient-specific anatomical and functional data, enabling personalized treatment planning^[12]^. Machine learning models can predict procedural success, identify potential complications, and assist in device selection, thereby improving clinical decision-making^[13]^. These tools are particularly valuable in complex cases where traditional imaging may provide limited guidance.

Advanced imaging technologies, such as three-dimensional echocardiography, cardiac MRI, and CT, are increasingly used to enhance procedural precision. These modalities provide detailed visualization of the tricuspid valve anatomy, surrounding structures, and the extent of regurgitation, facilitating accurate device placement^[14]^. Real-time imaging integration during procedures, such as intracardiac echocardiography (ICE) or fusion imaging, allows operators to navigate complex anatomies with greater confidence and precision^[15]^. The development of holographic imaging and virtual reality systems could further enhance operator training and procedural planning in the future.

Moreover, combining AI and imaging opens new frontiers for patient monitoring and follow-up. Remote monitoring systems equipped with AI algorithms can analyze data from wearable devices and implanted sensors, providing early detection of complications such as device malposition or worsening heart failure^[16]^. These innovations improve patient outcomes and reduce the burden on healthcare systems by enabling timely interventions.

Interdisciplinary collaboration is another critical aspect of advancing percutaneous tricuspid valve interventions. Successfully executing these procedures often requires a heart team approach involving cardiologists, interventionalists, imaging specialists, and surgeons. Efforts to streamline communication and decision-making among these stakeholders are essential for optimizing outcomes^[17]^. Training programs and simulation-based learning modules are being developed to equip clinicians with the skills needed to perform these complex procedures, further enhancing the adoption and success of percutaneous approaches.

## Implications for cardiac care

Percutaneous interventions, characterized by their minimally invasive nature, have redefined the treatment landscape for TVD. Unlike open surgical procedures, these interventions significantly reduce the procedural risk associated with advanced age, frailty, and comorbidities – factors commonly seen in patients with TR^[56]^. As a result, these procedures have expanded treatment eligibility to previously high-risk or inoperable populations. Patients with severe symptomatic TR, who were often managed with palliative medical therapy due to the prohibitive risks of surgery, now have access to interventions such as edge-to-edge repair systems (e.g., the TriClip) and transcatheter valve replacement devices (e.g., EVOQUE)^[15]^.

Studies have shown that these technologies improve clinical outcomes, including reductions in hospitalizations, symptomatic relief, and improved quality of life^[15]^. The emergence of percutaneous interventions has necessitated a collaborative approach among cardiologists, interventionalists, and cardiothoracic surgeons, fundamentally altering traditional roles within the cardiac care team. While cardiologists remain instrumental in diagnosing and managing TVD, interventional cardiologists now perform percutaneous procedures, requiring a high degree of technical skill and knowledge of device-specific applications. Simultaneously, cardiac surgeons play a crucial role in patient selection and addressing cases where surgical intervention remains optimal^[57]^.

This multidisciplinary approach fosters shared decision-making and leverages the strengths of each specialty, ensuring personalized and comprehensive care for patients. For example, in high-risk cases, joint heart team meetings are often convened to discuss the feasibility of percutaneous intervention versus surgery or optimal medical management. This collaborative model is particularly important given the complexity of tricuspid valve anatomy and the heterogeneity of patients with TVD^[6]^.

Interdisciplinary collaboration also extends to imaging specialists, who provide essential support during percutaneous interventions. Advanced imaging modalities, such as 3D echocardiography and cardiac CT, are integral to pre-procedural planning, intra-procedural guidance, and post-procedural follow-up^[58]^. These imaging techniques allow precise assessment of tricuspid valve anatomy, TR severity, and patients’ suitability for specific devices. For instance, real-time 3D echocardiography guides edge-to-edge repair, ensuring accurate placement of the device and minimizing procedural complications (see Table 1)^[59]^.

The seamless integration of imaging expertise into the cardiac care team underscores the importance of cross-disciplinary communication and training in successfully executing these interventions. The economic and logistical considerations associated with percutaneous interventions further highlight their transformative impact on cardiac care. While the upfront costs of these technologies can be substantial, their potential to reduce long-term healthcare expenditures is significant. Severe TR is associated with high rates of recurrent hospitalizations, particularly for heart failure exacerbations, which impose a considerable financial burden on healthcare systems^[60]^. Percutaneous interventions can decrease hospital readmissions and associated costs by alleviating symptoms and improving hemodynamics. For example, the cost-effectiveness of the TriClip system has been demonstrated in studies showing a reduction in heart failure hospitalizations and improved functional status, leading to better resource utilization over time^[61]^.

Moreover, the logistical feasibility of percutaneous interventions allows their adoption in a broader range of healthcare settings, including those with limited surgical facilities. Unlike open-heart surgery, which requires advanced surgical infrastructure and intensive care resources, percutaneous procedures can often be performed in catheterization laboratories with shorter hospital stays and faster recovery times^[62]^. This not only enhances patient accessibility but also alleviates the strain on surgical services, particularly in regions with limited healthcare infrastructure. However, these advantages must be weighed against the challenges of training and maintaining a workforce skilled in these advanced techniques and ensuring equitable access to devices and expertise across diverse healthcare systems^[63]^.

Despite these advancements, the widespread adoption of percutaneous interventions in TVD remains contingent on addressing several challenges. One of the key hurdles is the paucity of long-term data on the durability and effectiveness of these devices. While early clinical trials have demonstrated promising results, extended follow-up studies are necessary to establish long-term safety and efficacy^[64]^. Additionally, the regulatory approval process for novel devices can be lengthy, delaying patient availability. Healthcare providers and policymakers must work collaboratively to streamline these processes while maintaining rigorous safety standards^[65]^.

Another consideration is the need for robust healthcare policies to ensure equitable access to percutaneous interventions. The high upfront costs of these devices may limit their availability in low- and middle-income countries, exacerbating global disparities in cardiac care^[66]^. Innovative funding mechanisms, such as public-private partnerships and value-based reimbursement models, could help mitigate these barriers, ensuring that the benefits of these technologies reach underserved populations. Furthermore, healthcare systems must invest in training programs to build a skilled workforce capable of performing these complex procedures, particularly in regions with limited expertise in interventional cardiology^[67]^.

## Patient-centered perspectives

Percutaneous interventions provide patients with minimally invasive alternatives to open surgery, which is often contraindicated in those with advanced age or multiple comorbidities. Unlike surgical repair or replacement, these procedures typically involve shorter hospital stays, reduced recovery times, and fewer perioperative complications. For patients, this translates into an earlier return to daily activities and an overall improvement in functional capacity.

Clinical studies evaluating percutaneous devices such as the TriClip (Abbott) or the EVOQUE TVVR (Edwards Lifesciences) have consistently demonstrated significant reductions in TR severity^[68]^. These outcomes have been associated with improved New York Heart Association (NYHA) functional class scores, enhanced six-minute walk test distances, and a substantial decrease in heart failure-related hospitalizations^[69]^.

Patient-reported outcomes (PROs) are increasingly emphasized as critical endpoints in cardiovascular care. In the context of tricuspid valve interventions, PROs provide invaluable insights into the subjective benefits patients experience following the procedure. Studies have shown that patients who undergo percutaneous tricuspid interventions report higher satisfaction rates, improved emotional well-being, and greater confidence in managing their symptoms^[69-71]^.

For instance, a recent analysis of PROs from the TRILUMINATE trial revealed significant improvements in health-related QoL scores measured using the Kansas City Cardiomyopathy Questionnaire (KCCQ). Patients reported fewer physical limitations, better symptom management, and enhanced social functioning post-intervention^[72]^. These findings underscore the pivotal role of addressing the clinical outcomes and the lived experiences of patients in advancing cardiac care.

While the procedural benefits are evident, patient education is crucial in optimizing outcomes and ensuring long-term success. Understanding the disease’s nature, the intervention’s rationale, and the expected outcomes fosters shared decision-making and enhances adherence to post-procedural care. Given the novelty of percutaneous tricuspid interventions, patients often harbor anxieties about the risks and uncertainties associated with these procedures^[72,73]^. Addressing these concerns through comprehensive pre-procedural counseling can alleviate fear, enhance trust, and improve patient experience.

Educational initiatives must also extend beyond the procedure to encompass the importance of lifestyle modifications, adherence to medical therapies, and regular follow-up care. The psychosocial aspects of managing TVD with new technologies cannot be overstated. Chronic diseases such as TR often impose a significant psychological burden on patients, manifesting as anxiety, depression, or feelings of helplessness^[74,75]^. Introducing percutaneous interventions represents a shift in the narrative for many of these patients, offering hope and empowerment.

Successful interventions not only improve physical symptoms but also contribute to enhanced mental well-being. Several qualitative studies have documented patients’ renewed optimism and a sense of normalcy following percutaneous tricuspid procedures, highlighting the profound psychosocial impact of these advancements^[76]^.

Nonetheless, challenges remain in ensuring equitable access to these cutting-edge technologies. Socioeconomic factors, healthcare disparities, and limited availability of expertise in certain regions may impede the widespread adoption of percutaneous interventions. Policymakers and healthcare systems must prioritize strategies that address these barriers to provide patients across diverse settings with equal opportunities to benefit from these transformative therapies^[76]^.

## Concluding remarks

Percutaneous interventions in TVD represent a transformative leap in cardiac care, bridging critical gaps that traditional surgical and medical therapies left. As innovation drives new techniques and devices, these minimally invasive approaches redefine treatment paradigms and offer hope to patients previously considered ineligible for interventions. While challenges remain, the rapid evolution of this field underscores its potential to improve outcomes and reshape the future of valvular heart disease management.

Clinicians, researchers, and policymakers must unite in championing this progress. We call upon cardiovascular societies, funding bodies, and industry partners to prioritize research, training, and equitable access to percutaneous tricuspid therapies. Hospitals and institutions should invest in multidisciplinary heart teams and adopt standardized protocols to ensure safe and effective implementation.

The time to act is now. Let us commit to driving this progress – together, advancing care, improving survival, and setting a new standard for the management of tricuspid valve disease.

## Data Availability

This published article and its supplementary information files include all data generated or analyzed during this study.

## References

[1] Bishop MA Borsody K Percutaneous Tricuspid Valve Repair. [Updated 2023 Jun 21]. In: StatPearls]. Treasure Island (FL): StatPearls Publishing; 2025. Available from: https://www.ncbi.nlm.nih.gov/sites/books/NBK564430 33232088

[2] Asmarats L Taramasso M Rodés-Cabau J. Tricuspid valve disease: diagnosis, prognosis and management of a rapidly evolving field. Nat Rev Cardiol 2019;16:538–54. 30988448 10.1038/s41569-019-0186-1

[3] Campelo-Parada F Lairez O Carrié D. Percutaneous treatment of the tricuspid valve disease: new hope for the “Forgotten”. Valve Rev Esp Cardiol (Engl Ed) 2017;70:856–66. 28645836 10.1016/j.rec.2017.05.010

[4] Fam NP Connelly KA Hammerstingl C. Transcatheter tricuspid repair with mitraclip for severe primary tricuspid regurgitation. J Invasive Cardiol 2016;28:E223–E224. 27922813

[5] Zhu QM Berry N. Tricuspid regurgitation: disease state and advances in percutaneous therapy. Eur Cardiol 2023;18:e55. 37860699 10.15420/ecr.2023.09 PMC10583156

[6] Oh JS Kim GY Kim SH. Novel percutaneous technique for creation of porcine model of tricuspid regurgitation via two routes. J Int Med Res 2024;52:3000605241233524. 38497134 10.1177/03000605241233524 PMC10946071

[7] Minciunescu A Emaminia A. Contemporary evaluation and treatment of tricuspid regurgitation. Front Cardiovasc Med 2024;11:1350536. 38500755 10.3389/fcvm.2024.1350536 PMC10944863

[8] Otto CM Nishimura RA Bonow RO. 2020 ACC/AHA guideline for the management of patients with valvular heart disease: a report of the American College of Cardiology/American Heart Association joint committee on clinical practice guidelines. Circulation 2021;143:e72–227. 33332150 10.1161/CIR.0000000000000923

[9] Offen S Playford D Strange G. Adverse prognostic impact of even mild or moderate tricuspid regurgitation: insights from the National echocardiography database of Australia. J Am Soc Echocardiogr 2022;35:810–17. 35421545 10.1016/j.echo.2022.04.003

[10] Chorin E Rozenbaum Z Topilsky Y. Tricuspid regurgitation and long-term clinical outcomes. Eur Heart J Cardiovasc Imaging 2020;21:157–65. 31544933 10.1093/ehjci/jez216

[11] Kebed KY Addetia K Henry M. Refining severe tricuspid regurgitation definition by echocardiography with a new outcomes-based “massive” grade. J Am Soc Echocardiogr 2020;33:1087–94. 32651124 10.1016/j.echo.2020.05.007 PMC7955649

[12] Wang TKM Mentias A Akyuz K. Effect of tricuspid valve repair or replacement on survival in patients with isolated severe tricuspid regurgitation. Am J Cardiol 2022;162:163–69. 34903339 10.1016/j.amjcard.2021.08.069

[13] Dreyfus J Audureau E Bohbot Y. TRI-SCORE: a new risk score for in-hospital mortality prediction after isolated tricuspid valve surgery. Eur Heart J 2022;43:654–62. 34586392 10.1093/eurheartj/ehab679 PMC8843795

[14] Zhan Y Debs D Khan MA. Natural history of functional tricuspid regurgitation quantified by cardiovascular magnetic resonance. J Am Coll Cardiol 2020;76:1291–301. 32912443 10.1016/j.jacc.2020.07.036

[15] Estévez-Loureiro R Sánchez-Recalde A Amat-Santos IJ. 6-month outcomes of the TricValve system in patients with tricuspid regurgitation: the TRICUS EURO study. JACC Cardiovasc Interv 2022;15:1366–77. 35583363 10.1016/j.jcin.2022.05.022

[16] Mehr M Taramasso M Besler C. 1-year outcomes after edge-to-edge valve repair for symptomatic tricuspid regurgitation: results from the TriValve registry. JACC Cardiovasc Interv 2019;12:1451–61. 31395215 10.1016/j.jcin.2019.04.019

[17] Nickenig G Weber M Schüler R. Tricuspid valve repair with the Cardioband system: two-year outcomes of the multicentre, prospective TRI-REPAIR study. EuroIntervention 2021;16:e1264–71. 33046437 10.4244/EIJ-D-20-01107 PMC9724932

[18] Nickenig G Friedrichs KP Baldus S. Thirty-day outcomes of the Cardioband tricuspid system for patients with symptomatic functional tricuspid regurgitation: the TriBAND study. EuroIntervention 2021;17:809–17. 34031021 10.4244/EIJ-D-21-00300 PMC9724867

[19] Fam NP von Bardeleben RS Hensey M. Transfemoral transcatheter tricuspid valve replacement with the EVOQUE system: a multicenter, observational, first-in-human experience. JACC Cardiovasc Interv 2021;14:501–11. 33582084 10.1016/j.jcin.2020.11.045

[20] Webb JG Chuang AM Meier D. Transcatheter tricuspid valve replacement with the EVOQUE system: 1-year outcomes of a multicenter, first-in-human experience. JACC Cardiovasc Interv 2022;15:481–91. 35272772 10.1016/j.jcin.2022.01.280

[21] Kodali S Hahn RT George I. Transfemoral tricuspid valve replacement in patients with tricuspid regurgitation. JACC Cardiovasc Interv 2022;15:471–80. 35272771 10.1016/j.jcin.2022.01.016

[22] Lurz P Stephan von Bardeleben R Weber M. Transcatheter edge-to-edge repair for treatment of tricuspid regurgitation. J Am Coll Cardiol 2021;77:229–39. 33478646 10.1016/j.jacc.2020.11.038

[23] Kodali S Hahn RT Eleid MF. Feasibility study of the transcatheter valve repair system for severe tricuspid regurgitation. J Am Coll Cardiol 2021;77:345–56. 33509390 10.1016/j.jacc.2020.11.047

[24] Sorajja P Whisenant B Hamid N. Transcatheter repair for patients with tricuspid regurgitation. N Engl J Med 2023;388:1833–42. 36876753 10.1056/NEJMoa2300525

[25] Rogers JH. Functional tricuspid regurgitation: percutaneous therapies needed. JACC Cardiovasc Interv 2015;8:492–94. 25703876 10.1016/j.jcin.2014.11.013

[26] Hahn RT Nabauer M Zuber M. Intraprocedural imaging of transcatheter tricuspid valve interventions. JACC Cardiovasc Imaging 2019;12:532–53. 30846126 10.1016/j.jcmg.2018.07.034

[27] Cevasco M Shekar PS. Surgical management of tricuspid stenosis. Ann Cardiothorac Surg 2017;6:275–82. 28706872 10.21037/acs.2017.05.14 PMC5494417

[28] Khan MS Baqi A Tahir A. National estimates for the percentage of all readmissions with demographic features, morbidity, overall and gender-specific mortality of transcutaneous versus open surgical tricuspid valve replacement/repair. Cardiol Res 2024;15:223–32. 39205967 10.14740/cr1625 PMC11349133

[29] Harb SC Spilias N Griffin BP. Surgical repair for primary tricuspid valve disease: individualized surgical planning with 3-dimensional printing. JACC Case Rep 2020;2:2217–22. 34317143 10.1016/j.jaccas.2020.09.047 PMC8299861

[30] Samim D Praz F Cochard B. Natural history and mid-term prognosis of severe tricuspid regurgitation: a cohort study. Front Cardiovasc Med 2023;9:1026230. 36698931 10.3389/fcvm.2022.1026230 PMC9870052

[31] Henning RJ. Tricuspid valve regurgitation: current diagnosis and treatment. Am J Cardiovasc Dis 2022;12:1–18. 35291509 PMC8918740

[32] Barker CM Goel K. Transcatheter tricuspid interventions: past, present, and future. Methodist Debakey Cardiovasc J 2023;19:57–66. 37213880 10.14797/mdcvj.1250 PMC10198234

[33] Barker CM Cork DP McCullough PA. Comparison of survival in patients with clinically significant tricuspid regurgitation with and without heart failure (From the Optum Integrated File). Am J Cardiol 2021;144:125–30. 33385352 10.1016/j.amjcard.2020.12.070

[34] Barker CM Cork DP McCullough PA. Healthcare utilization in clinically significant tricuspid regurgitation patients with and without heart failure. J Comp Eff Res 2021;10:29–37. 33174767 10.2217/cer-2020-0198

[35] Cork DP McCullough PA Mehta HS. The economic impact of clinically significant tricuspid regurgitation in a large, administrative claims database. J Med Econ 2020;23:521–28. 31952454 10.1080/13696998.2020.1718681

[36] Sun YP O’Gara PT. Epidemiology, anatomy, pathophysiology and clinical evaluation of functional tricuspid regurgitation. Minerva Cardioangiol 2017;65:469–79. 28398019 10.23736/S0026-4725.17.04398-5

[37] Silbiger JJ. Atrial functional tricuspid regurgitation: an underappreciated cause of secondary tricuspid regurgitation. Echocardiography 2019;36:954–57. 30919501 10.1111/echo.14327

[38] Kalra A Uberoi AS Latib A. Emerging transcatheter options for tricuspid regurgitation. Methodist Debakey Cardiovasc J 2017;13:120–25. 29743996 10.14797/mdcj-13-3-120 PMC5935195

[39] Prihadi EA Delgado V Leon MB. Morphologic types of tricuspid regurgitation: characteristics and prognostic implications. JACC Cardiovasc Imaging 2019;12:491–99. 30846123 10.1016/j.jcmg.2018.09.027

[40] Beg F Dadu RT Reardon MJ. Simultaneous transfemoral mitral and tricuspid valve in ring implantation: first case report with Edwards Sapien 3 Valve. Methodist Debakey Cardiovasc J 2019;15:149–51. 31384379 10.14797/mdcj-15-2-149 PMC6668740

[41] Davidson LJ Davidson CJ. Transcatheter treatment of valvular heart disease: a review. JAMA 2021;325:2480–94. 34156404 10.1001/jama.2021.2133

[42] Hahn RT Kodali S Fam N. Early Multinational experience of transcatheter tricuspid valve replacement for treating severe tricuspid regurgitation. JACC Cardiovasc Interv 2020;13:2482–93. 33153565 10.1016/j.jcin.2020.07.008

[43] Nishimura RA Otto CM Bonow RO. 2014 AHA/ACC Guideline for the Management of Patients With Valvular Heart Disease. A Report of the American College of Cardiology/American Heart Association Task Force on Practice Guidelines. J Am Coll Cardiol 2014;63:e57–185. 24603191 10.1016/j.jacc.2014.02.536

[44] Taramasso M Hahn RT Alessandrini H. The International Multicenter TriValve Registry: which patients are undergoing transcatheter tricuspid repair? JACC Cardiovasc Interv 2017;10:e57–185. 28982563 10.1016/j.jcin.2017.08.011

[45] Taramasso M Gavazzoni M Pozzoli A. Tricuspid regurgitation: predicting the need for intervention, procedural success, and recurrence of disease. JACC Cardiovasc Imaging 2019;12:605–21. 30947904 10.1016/j.jcmg.2018.11.034

[46] Taramasso M Benfari G Vanc der Bijl P. Transcatheter versus medical treatment of patients with symptomatic severe tricuspid regurgitation. J Am Coll Cardiol 2019;74:2998–3008. 31568868 10.1016/j.jacc.2019.09.028

[47] Nickenig G Weber M Lurz P. Transcatheter edge-to-edge repair for reduction of tricuspid regurgitation: 6-month outcomes of the TRILUMINATE single-arm study. Lancet 2019;394:2002–11. 31708188 10.1016/S0140-6736(19)32600-5

[48] Fam NP Braun D von Bardeleben RS. Compassionate use of the PASCAL transcatheter valve repair system for severe tricuspid regurgitation: a multicenter, observational, first-in-human experience. JACC Cardiovasc Interv 2019;12:2488–95. 31857018 10.1016/j.jcin.2019.09.046

[49] Praz F Muraru D Kreidel F. Transcatheter treatment for tricuspid valve disease. EuroIntervention 2021;17:791–808. 34796878 10.4244/EIJ-D-21-00695 PMC9724890

[50] Elgharably H Harb SC Kapadia S. Transcatheter innovations in tricuspid regurgitation: navigate. Prog Cardiovasc Dis 2019;62:493–95. 31707062 10.1016/j.pcad.2019.11.004

[51] Hahn RT George I Kodali SK. Early single-site experience with transcatheter tricuspid valve replacement. JACC Cardiovasc Imaging 2019;12:416–29. 30553658 10.1016/j.jcmg.2018.08.034

[52] Aoi S Wiley J Ho E. Transcatheter tricuspid valve implantation with the Cardiovalve system. Future Cardiol 2021;17:963–69. 33512242 10.2217/fca-2020-0181

[53] Barreiro-Perez M Estevez-Loureiro R Baz JA. Cardiovalve transfemoral tricuspid valve replacement assisted with CT-fluoroscopy fusion imaging. JACC Cardiovasc Interv 2022;15:e197–e199. 36075653 10.1016/j.jcin.2022.06.023

[54] Lu FL Ma Y An Z. First-in-man experience of transcatheter tricuspid valve replacement with LuX-valve in high-risk tricuspid regurgitation patients. JACC Cardiovasc Interv 2020;13:1614–16. 32646711 10.1016/j.jcin.2020.03.026

[55] Hufnagel CA. Aortic plastic valvular prosthesis. Bull Georgetown Univ Med Cent 1951;4:128–30. 24539965

[56] Figulla HR Kiss K Lauten A. Transcatheter interventions for tricuspid regurgitation - heterotopic technology: tricValve. EuroIntervention 2016;12:Y116–8. 27640022 10.4244/EIJV12SYA32

[57] O’Neill BP Negrotto S Yu D. Caval valve implantation for tricuspid regurgitation: insights from the United States Caval Valve Registry. J Invasive Cardiol 2020;32:470–75. 33087585 10.25270/jic/20.00371

[58] Avenatti E Barker CM Little SH. Tricuspid regurgitation repair with a MitraClip device: the pivotal role of 3D transoesophageal echocardiography. Eur Heart J Cardiovasc Imaging 2017;18:380. 28065911 10.1093/ehjci/jew311

[59] Zoltowska DM Maaliki N Al-Turk B. A valve-in-valve approach to manage severe bioprosthetic tricuspid valve stenosis. J Geriatr Cardiol 2021;18:400–02. 34149828 10.11909/j.issn.1671-5411.2021.05.002 PMC8185438

[60] Saad Shaukat MH Stys J Stys A. Transcatheter tricuspid valve-in-valve implantation for very early bioprosthetic tricuspid stenosis secondary to pacemaker lead entrapment: a case report. Eur Heart J Case Rep 2022;6:ytac251. 35799681 10.1093/ehjcr/ytac251 PMC9257794

[61] Baumgartner H Falk V Bax JJ. 2017 ESC/EACTS Guidelines for the management of valvular heart disease. Eur Heart J 2017;38: 2739–91. 28886619 10.1093/eurheartj/ehx391

[62] Marquis-Gravel G Bouchard D Perrault LP. Retrospective cohort analysis of 926 tricuspid valve surgeries: clinical clinical and hemodynamic outcomes with propensity score analysis. Am Heart J 2012;163:851–58. 22607864 10.1016/j.ahj.2012.02.010

[63] Otto CM Nishimura RA Bonow RO. 2020 ACC/AHA Guideline for the Management of Patients With Valvular Heart Disease: executive Summary: a Report of the American College of Cardiology/American Heart Association Joint Committee on Clinical Practice Guidelines. Circulation 2021;143:e35–e71. 33332149 10.1161/CIR.0000000000000932

[64] Dvir D Bourguignon T Otto CM. Standardized definition of structural valve degeneration for surgical and transcatheter bioprosthetic aortic valves. Circulation 2018;137:388–99. 29358344 10.1161/CIRCULATIONAHA.117.030729

[65] Koziarz A Makhdoum A Butany J. Modes of bioprosthetic valve failure: a narrative review. Curr Opin Cardiol 2020;35:123–32. 31972604 10.1097/HCO.0000000000000711

[66] Taramasso M Hahn RT Alessandrini H. The International Multicenter TriValve Registry. J Am Coll Cardiol Intventions 2017; 10:1982–90.

[67] Shivaraju A Michel J Frangieh AH. Transcatheter aortic and mitral valve-in-valve implantation using the Edwards Sapien 3 Heart Valve. J Am Heart Assoc 2018;7:e007767. 29982230 10.1161/JAHA.117.007767 PMC6064864

[68] McElhinney DB Cabalka AK Aboulhosn JA. Valve-in-Valve International Database (VIVID) registry. Transcatheter tricuspid valve-in-valve implantation for the treatment of dysfunctional surgical bioprosthetic valves: an international, multicenter registry study. Circulation 2016;133:1582–93. 26994123 10.1161/CIRCULATIONAHA.115.019353

[69] Lamprea-Montealegre JA Oyetunji S Bagur R. Valvular heart disease in relation to race and ethnicity: JACC focus seminar 4/9. J Am Coll Cardiol 2021;78:2493–504. 34886971 10.1016/j.jacc.2021.04.109

[70] CDC. Valvular heart disease (last reviewed 2019). Available online at: Accessed 19 September 2023). https://www.cdc.gov/heartdisease/valvular_disease.htm#:~:text=About%202

[71] Wang N Fulcher J Abeysuriya N. Tricuspid regurgitation is associated with increased mortality independent of pulmonary pressures and right heart failure: a systematic review and meta-analysis. Eur Heart J 2019;40:476–84. 30351406 10.1093/eurheartj/ehy641

[72] Topilsky Y Maltais S Medina Inojosa J. Burden of tricuspid regurgitation in patients diagnosed in the community setting. JACC Cardiovasc Imaging 2019;12:433–42. 30121261 10.1016/j.jcmg.2018.06.014

[73] Virani SS Alonso A Aparicio HJ. Heart disease and stroke statistics-2021 update: a report from the American Heart Association. Circulation 2021;143:e254–743. 33501848 10.1161/CIR.0000000000000950 PMC13036842

[74] Vahanian A Beyersdorf F Praz F. 2021 ESC/EACTS guidelines for the management of valvular heart disease. Eur Heart J 2022;43: 561–632. 34453165 10.1093/eurheartj/ehab395

[75] Batchelor W Emaminia A. Tricuspid regurgitation and right heart failure: “it all begins and ends with the RV.” JACC Heart Fail 2020;8: 637–39. 32731946 10.1016/j.jchf.2020.04.013

[76] Enriquez-Sarano M Messika-Zeitoun D Topilsky Y. Tricuspid regurgitation is a public health crisis. Prog Cardiovasc Dis 2019;62: 447–51. 31707061 10.1016/j.pcad.2019.10.009

